# The Achievements and Challenges of Mesenchymal Stem Cell-Based Therapy in Inflammatory Bowel Disease and Its Associated Colorectal Cancer

**DOI:** 10.1155/2020/7819824

**Published:** 2020-03-18

**Authors:** Dickson Kofi Wiredu Ocansey, Wei Qiu, Jingyan Wang, Yongmin Yan, Hui Qian, Xu Zhang, Wenrong Xu, Fei Mao

**Affiliations:** ^1^Key Laboratory of Medical Science and Laboratory Medicine of Jiangsu Province, School of Medicine, Jiangsu University, Zhenjiang, 212013 Jiangsu, China; ^2^Directorate of University Health Services, University of Cape Coast, Cape Coast, Ghana; ^3^Jiangning Hospital of Nanjing, Nanjing, 211100 Jiangsu, China

## Abstract

Approximately 18.1 × 10^6^ new cases of cancer were recorded globally in 2018, out of which 9.6 million died. It is known that people who have Inflammatory Bowel Disease (IBD) turn to be prone to increased risks of developing colorectal cancer (CRC), which has global incident and mortality rates of 10.2% and 9.2%, respectively. Over the years, conventional treatments of IBD and its associated CRC have been noted to provide scarce desired results and often with severe complications. The introduction of biological agents as a better therapeutic approach has witnessed a great deal of success in both experimental and clinical models. With regard to mesenchymal stem cell (MSC) therapy, the ability of these cells to actively proliferate, undergo plastic differentiation, trigger strong immune regulation, exhibit low immunogenicity, and express abundant trophic factors has ensured their success in regenerative medicine and immune intervention therapies. Notwithstanding, MSC-based therapy is still confronted with some challenges including the likelihood of promoting tumor growth and metastasis, and possible overestimated therapeutic potentials. We review the success story of MSC-based therapy in IBD and its associated CRC as documented in experimental models and clinical trials, examining some of the challenges encountered and possible ways forward to producing an optimum MSC therapeutic imparts.

## 1. Introduction

Over the years, IBD treatment has chiefly been surgical operations and drug therapy administration. While the former is prone to high risks due to its invasiveness, the latter is not capable of eradicating the underlying danger [1]. These conventional therapeutic methods have low clinical remission rates for IBD (20%–30%), with a remission rate reaching roughly 50% when combined therapies are applied. In the same way, efficient treatment options for colitis-associated CRC have been highly difficult to arrive at; in many cases, clients were taken through cancer lesion removal via surgical resections with later support from other treatment options like radiotherapy and chemotherapy [2].

For some years now, development in medicine has applied human stem cell therapy to treat tissue-related conditions including IBD. The application of induced pluripotent stem cells, MSCs, and embryonic stem cells has indicated encouraging outcomes whereby these cells proliferate and differentiate resulting in the replacement/repair of tissues [3]. MSCs capably respond to inflammatory cytokines and highly interact with the adaptive as well as innate immune components by secreting immunomodulatory particles that control inflammation development via influencing T cell, dendritic cell, NK cell, macrophage, and B cell [4]. MSCs in their functions produce multiplicity of substances in a paracrine fashion that results in their desired effects. Among the several chemicals secreted are cytokines, growth factors, and extracellular vesicles like exosomes [5]. These vesicles, for some time now, are identified as efficient transporters in intercellular communications, within the eukaryotic and prokaryotic organism. This property has been attributed to their capability to transport nucleic acids, lipids, and proteins, hence imparting several pathological as well as physiological functionalities or behaviors of parent cells and recipient cells including the development and repair of injured tissues [6, 7].

It is crucially important to examine the documented results of MSC therapeutic application in both the experimental and the clinical trial settings of IBD and its associated CRC, considering the successes achieved and challenges confronted. This will give room for capitalizing on the achievements and setting possible ways of brazing out the challenges towards producing an optimum MSC therapeutic influence. We will also review exosomes from MSCs as cell-free therapy and whether it could bridge some of the gaps seen in MSC-based therapy in IBD.

## 2. Characteristics of Mesenchymal Stem Cells

MSCs, as none hematopoietic precursor cells, possess several properties including their capability to differentiate to produce different kinds of cells like adipocytes, osteocytes, fibroblasts, and neurocytes [8]. They are resident within bone marrows and found in certain other tissues like umbilical cord blood, adipose, and dental pulp and assist homeostasis in healthy tissues in the process of wound healing and regeneration. While they do not express CD31 (endothelial marker) and CD45 (hematopoietic marker), they rather highly express CD90, CD73, and CD105 [9]. Among the classical properties that render these cells highly appealing as immunomodulatory substances are their capacity of homing within injury and inflammatory sites and secreting cytokines and/or growth factors to enhance repair, diminish inflammatory activities, or differentiate into the different types of damaged tissues [10]. The ability of MSCs to quickly interact with their surroundings and get stimulated also enhances their functionality as anti-inflammatory agents. Again, proinflammatory cytokines, such as IL-1*β*, TNF-*α*, IFN-*γ*, and IL-6, adequately impart the immune-suppressive abilities of MSCs [11].

## 3. Influence of Mesenchymal Stem Cells

**Figure 1:**
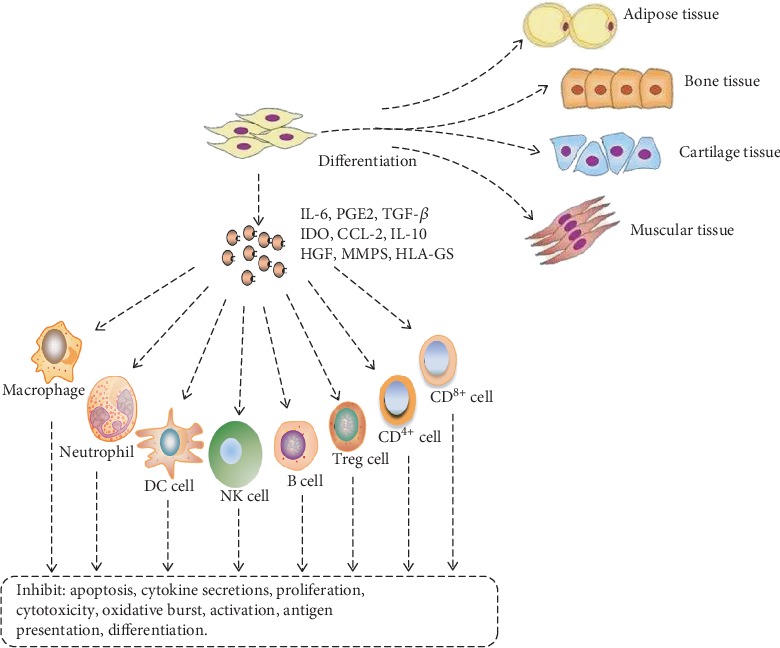
The general effects of MSCs grouped under two major mechanisms: direct cellular differentiation of recruited MSCs (into adipose, bone, cartilage, and muscle tissues) to replace damaged cells, and preparation of the inflammatory environment by MSC-secreted cytokines as they influence the host immune system. IL-6: interleukin-6; PGE2: prostaglandin E2; TGF-*β*: transforming growth factor-*β*; IDO: indoleamine 2,3-dioxygenase; CCL-2: C-C motive chemokine ligand 2; IL-10: interleukin-10; HGF: hepatocyte growth factor; MMPs: Matrix Metallopeptidases; HLA-Gs: Human Leukocyte Antigen-Gs.

The influence of MSC can be grouped into two main mechanisms. The first mechanism is the differentiation of MSCs which have been recruited as actively functional cells to replenish injured cells. This enhances the repair of damages to tissues like muscles, bones, and cartilages. The second mechanism is the involvement of MSCs in the preparation of the microenvironment (as a consequence of their stimulation by inflammatory agents), via secreting immune-regulatory particles that control immune system cells (see Figure 1). In this mechanism, they produce a significant quantity of cytokines including exosomes that inhibit apoptosis, trigger angiogenesis, stimulate stem cell differentiation, hinder oxidative reaction, and foster extracellular matrix remodeling [9].

### 3.1. Interactions with the Innate Immune System

Macrophages and DCs play critical roles in the initiation of chronic inflammation and pathogenesis of IBD as adequately expounded by Steinbach et al. [12]. Monocytes and CD68+ macrophages recruited into the lamina of the intestinal inflammatory tissues decrease the expression levels of tight junction proteins, leading to compromised intestinal epithelial cell barrier integrity and reduced function, creating accelerated disease progression. The macrophage-mediated effects are primarily through TNF-*α*, whereas that of monocytes are through IL-1*β* and IL-18 [13]. Macrophages could also be considered the first line of defense against tumors on the basis that they are capable of rapidly colonizing and secreting cytokines that activate other components of innate immunity like DC and NK cell and are capable of phagocytosing a lifeless tumor cell as well as presenting antigens associated with tumors to CD8+ T cells [14]. Available data indicates that, by utilizing their communication with macrophages, MSCs capably enhance their therapeutic effects by balance between M1 and M2 macrophages, as well as their tumor-promoting influence within tumor microenvironment (TME) [15]. In these interactions, macrophages could be polarized to an M1 phenotype which express inflammatory cytokines, nitrogen, and reactive oxygen species or an M2 phenotype that participates in inflammatory and tissue remodeling suppressions [16]. In expounding the mechanism involved in MSC and macrophage interaction in ameliorating colitis, Song and colleagues report that both human and canine adipose tissue-derived MSCs administered intraperitoneally secrete TSG-6 (tumor necrosis factor-*α*-induced gene/protein 6) which induces macrophage phenotypic switch from M1 to M2 [17, 18]. It is also reported that human umbilical cord-derived MSCs transfected with miR148b-5p mimics attenuate IBD via reducing the expression of 15-lox-1 in macrophages. The inhibitory effect of miR148b-5p on 15-lox-1 expression in macrophages results in enhanced colonic tissue repair [19]. In our recent review article on the crosstalk between MSCs and macrophages, we highlighted some of the mechanisms involved in MSC influence in activating macrophages to ameliorate IBD. This included M2 macrophage polarization via the TGF-*β* signaling pathway, alternative activation of macrophages via galectin-3 inhibition, and MSC pretreatment with IL-1 that inhibited CD11c+ M1 macrophages [20]. On the basis of this, M2 phenotype macrophages may serve as vital targets on the phase of new therapy development and adjunct to enhance effectiveness of existing or evolving ones [15]. Again, identifying specific features produced through MSCs within the TME responsible for the induction of M2 phenotypes would even be a high merit and a possible breakthrough in the development of cancer immunotherapy.

DCs and NK cells are involved in IBD pathogenesis [12], contribute significantly to antitumor immune reactions [21], and are known to be vital components of the colorectal TME. A lot of investigations have shown the fact that MSCs capably suppress maturation, cytokine secretion, and proliferation of DCs, NK cells, and T cells through the mediation of particles such as PGE_2_ and IDO (indoleamine 2,3-dioxygenase) which are produced via MSCs as they react to stimuli inflammation within TME of the colon [22]. Coculture of MSCs with DCs results in a decreased expression of IFN-*γ*, CD11c, CD80, CD86, IL-6, and TNF-*α* but an increased expression of CD11b, IL-10, and TGF-*β*. Administration of the MSC-DCs in DSS-induced colitis mice causes colon tissue IL-6, IFN-*γ*, and TNF-*α* to decrease while Foxp3, IL-10, and TGF-*β* increase. This implicates that MSCs differentiate DCs into regulatory DCs, which ameliorate colitis [23] and also suppress inflammatory phenotype of DCs in a galectin-3-dependent manner [24]. It is also documented that the regulatory DCs further trigger the production of Tregs to enhance the anti-inflammatory effect of MSCs in immune disorders. The high endocytosis capacity, low immunogenicity, and strong immunomodulatory effects of MSC-DCs are mediated through TGF-*β*1 and Treg cells via the efficient generation of CD4+CD25+Foxp3+ Treg cells from CD4+CD25-Foxp3-T cells [25]. Upregulated expression of IL-10 and Treg cells by human umbilical cord-derived MSCs has been associated with increased activation of the NOD2-RIP2 pathway and prolonged production of PGE2, which also inhibits the proliferation of mononuclear cells to attenuate colitis [26].

The interaction between MSCs and NK cells is highly complex in that, whereas certain factors (including the expression of activating NK cell receptor ligands on MSCs and the low MHC class I) make MSCs a natural target for activated NK cell killing, MSCs can also greatly alter NK cell phenotype and inhibit cytokine secretion and cytotoxicity against HLA class I expressing targets [27, 28]. Mechanisms known to aid MSCs to escape NK-mediated killing include IFN-*γ* pretreatment, Serine Protease Inhibitor 9 (SERPINB9), and modulation of TLRs. Whereas TLR3 stimulation protects MSCs from NK cell killing and promotes the immunosuppressive effect of MSCs on NK cells, TLR2 activation rather downregulates the immunosuppressive activity of MSCs [29, 30]. PGE2 and IDO are principal modulators of MSC-induced inhibition of NK cells [22].

Neutrophils defend host by killing invading microbes. However, they also produce detrimental effects in the TME via inhibiting apoptosis and promoting tumorigenesis [31]. It is known that neutrophils are defended by MSCs against apoptosis and that neutrophils triggered by tumor-associated MSC enhance ordinary MSCs to differentiate into cancer-associated fibroblast, hence promoting tumorigenesis [32]. On the other hand, normal MSCs reduce the number of neutrophils that bind to vascular endothelial cells, hence restraining the recruitment of these cells to inflammatory sites [33]. Additionally, MSC-secreted cytokines stimulate neutrophil chemotaxis and release of proinflammatory chemicals that partake in the stimulation and recruitment of phagocytic macrophage [34]. Furthermore, MSCs possess the ability to restrict secretion of proinflammatory chemokines by mast cell and limit the migratory and degranulation activities of these cells towards chemotactic factors. The mechanism involved is cell-to-cell contact resulting in the activation of inhibitory effects dependent on the upregulation of COX2 in MSCs, facilitated via the activation of EP4 receptors on mast cells [35].

### 3.2. Interactions with the Adaptive Immune System

Most of the work on MSC-mediated immunoregulations have centered on MSC influence on the proliferation as well as effector functionalities of T cells. One of such studies discovered that T cells activated through DCs, lymphocytes of peripheral blood, or phytohemagglutinin could inhibit the proliferation of MSCs through a contact-independent mechanism capable of being reversed by antibodies against HGF (hepatocyte growth factor) and TGF-*β*1 (transforming growth factor *β*1), thus the significant roles of such chemokines in MSC-linked immunoregulation [36]. Researchers have also shown IDO as vital in MSC-associated T cell suppressions, but in rodents' MSCs, nitric oxide is rather discovered as accountable for T cell suppressions while IDO seems expendable [37, 38]. In a DSS-induced colitis mouse model, adipose tissue-derived MSCs expressed PGE2 which induced FOXP3 mRNA expression. The upregulated expression of FOXP3+ Treg cells within the inflamed colonic tissue dampened the inflammation to resolve the colitis [39]. Similar finding is documented by Yang and colleagues who also demonstrated that the crosstalk between MSCs and T cells is mediated by PGE2. They also noted that the preconditioned human umbilical cord-derived MSCs elicited antiapoptotic influence through inducing the ERK pathway at the early stage of IBD development and as well inhibited TNF*α* and IL-2 while promoting IL-10 in T cells [40].

In a recent work on a rat colitis model, it was demonstrated that the immunoregulatory impacts of locally injected MSCs from adipose results in a recovered expression of Foxp3 and IL-10 mRNA levels in mesenteric lymph nodes [41]. Intraperitoneal administration of bone marrow MSCs formed aggregates within the peritoneal cavity of colitis mice. Analysis showed that the aggregates consisted of macrophages, B cells, and T cells, as well as immunomodulatory molecules like FOXP3, IL-10, TGF-*β*, CCL22, heme oxygenase-1, arginase type II, and TSG6. Subsequent injection of TSG6 increased Foxp3CD45+ cells but decreased CD45+ cells, neutrophils, and metalloproteinase activities in the mucosa, leading to reduced severity of colitis [42]. MSCs were also found to cause an increased expression of TGF-*β* resulting in an upregulation of Treg cells [43]. Similarly, increased expressions of Treg cells alongside reduction of cytotoxicity of NK cells and CD8+ T cells in experimental models have also been reported. In these reports, MSCs administered produced a substantial upregulation of TGF-*β*, IL-10, and IL-4 and a decreased expression of IFN-*γ* within the sera of tumor carrying mice. It also triggered a reduction in antitumor Th1 cytokines and upregulation in Th2 cytokines [43]. In a colon cancer experiment, the researchers found that MSCs that received the treatment of cytokine (TNF-*α*, IFN-*γ*) appeared effective enhancers of angiogenesis and ascribed the outcome to increase in vascular endothelial growth factor (VEGF) secretion in MSCs as a consequence of hypoxia-induced factor 1*α* (HIF-1*α*) signaling [44].

MSC and its derived exosomes are known to regulate the maturation, proliferation, and functional activation of B cells, T cells, and monocyte-derived dendritic cells via mechanisms that rely on cell contact and secreted molecules. In evaluating the mechanism involved in this interaction, Khare and colleagues studied the effect of bone marrow-derived MSC exosomes on B and T lymphocyte proliferation and activated peripheral blood mononuclear cells (PBMCs). They observe that proliferation of isolated T and B cells and activated PBMCs decreased by 23%, 18%, and 37%, respectively [45]. In another research, it was demonstrated that the modulatory effect of MSCs on B cells was partially mediated by soluble factors other than extracellular vesicles like exosomes [46]. In a clinical trial of MSC and infliximab-combined therapy, MSCs reduced autoreactive clone of B lymphocytes (CD19+CD5+) [47]. It was also reported that MSCs ameliorate B cell-mediated immune response and upregulate IL-10-expressing regulatory B cells in an EBI3-dependent manner [48]. Luk and colleagues further explain that immunological conditions determine the stimulatory action of MSCs on B cell. MSCs stimulate regulatory B induction under immunological quiescent conditions, whereas they inhibit B cell proliferation and maturation under inflammatory conditions via depletion of tryptophan [49].

## 4. Risk Factors of IBD and Its Associated CRC

**Figure 2:**
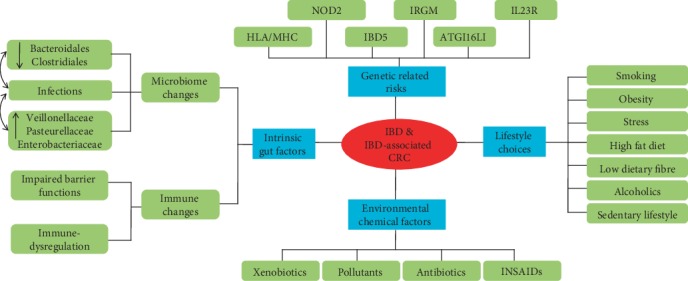
Risks factors associated with the unset and development of IBD/IBD-associated CRC. Though these factors mostly overlap, they can be put into four main categories which have many other subsets. NOD2: nucleotide-binding oligomerization domain-containing protein 2; HLA/MHC: Human Leukocyte Antigens/Major Histocompatibility Complex; IRGM: immunity-related GTPase family M protein; ATG16LI: authophagy-related 16-like gene; IL23R: interleukin 23 receptor; IBD5: Inflammatory Bowel Disease 5.

Although the manner and development of IBD is highly complicated, researchers have documented that hereditary and environmental influences play critical roles in stimulating intestinal immune system disorders leading to mucosal damages [3]. Chronic mucosal inflammatory damage is one of the key factors linked with the inception of carcinogenesis in an IBD patient. Even though several genetic alterations that result in sporadic CRCs also take place in patients with IBD-associated CRC, certain gene sequences as well as mutation frequencies differ between IBD-associated CRCs and sporadic CRCs [50]. Several other risk factors ranging from genetics [51], environmental, lifestyle, and intrinsic gut factors [52] also contribute to CRC occurrence in individuals having IBDs. Incidence of sporadic CRC in the family's history, active inflammations, degree as well as length of colonic disease, and coexistence of primary sclerosing cholangitis also prone individuals to CRC risks [52, 53]. These risk factors can be put into four main categories as shown in Figure 2.

## 5. Incident Rate

Cancer is among the top causes of mortality globally. According to global cancer statistics in 2018, there were approximately 18.1 × 10^6^ new incidents out of which 9.6 × 10^6^ deaths were recorded around the globe [54]. The report further states that 39.6% of both sexes would be diagnosed of a type of cancer at certain moment in their lifetimes. Patients with IBD are prone to greater risks of CRC development. IBD-associated CRC is responsible for the death of 10 to15% of patients with ulcerative colitis (UC), and it is also known to account for approximately 1 to 2% of all CRC cases. The incidence of colorectal cancer is third (10.2%) after lung cancer (11.6%) and breast cancer (10.6%) but second (9.2%) to lung cancer (18.4%) in terms of mortality according to global reports [54, 55]. Colorectal cancer is the third most common form of gastrointestinal cancers, with a report of more than one million newly diagnosed cases annually throughout the world [55].

## 6. Interaction between MSC, IBD, and IBD-Associated CRC

In the IBD microenvironment, there is imbalance of T cell subsets to include downregulated Treg cells. This dysregulation coupled with other immune, microbiome, and molecular factors lead to chronic inflammation. Chronic inflammation increases the risk of developing colitis-associated CRC by 2% after 10 years, 8% after 20 years, and 18% after 30 years of colitis [56]. Although the pathogenesis of colitis-associated CRC differs from that of sporadic CRC, they share several common characteristic mechanisms such as aneuploidy, mutations in APC (adenomatous polyposis coli) gene, DNA methylation, oncogene k-ras activation, microsatellite instability (MSI), COX-2 activation, tumor suppressor gene DCC/DPC4 mutation, and eventual loss of p53 functions [57]. One classical difference between the two is that, in sporadic cancers, the dysplastic precursor is the adenomatous polyp, but in IBD-associated CRC, the dysplasia can be localized, diffuse or multifocal [58–60]. MSC-based therapy is meant to resolve the colitis by modulating the immune response to restore balance (in immune cells, microbiome diversity, and composition) and repair intestinal tissue damages [61].

The two types of MSC transplant in IBD and its associated CRC are autologous and allogeneic transplant. In autologous transplantation, the patient receives his/her own MSCs (autologous MSCs) while in allogenic transplantation, the patient receives MSCs from a healthy donor (allogeneic MSCs). Mostly used MSCs in both clinical and experimental studies of IBD are human bone marrow-derived, adipose-derived, and umbilical cord-derived MSCs. Although bone marrow-derived MSCs (sometimes called the “gold standard”) are widely used, the invasive and painful nature of their acquisition limit their application in regenerative medicine. The higher convenience of obtaining adipose and umbilical cord MSCs has also increased their application across several studies [3, 62]. Other sources of MSCs applied in IBD and colitis-associated CRC are amniotic fluid [63], placenta [64], tonsil [65], amnion [66, 67], and endometrium regenerative cells [68], among others. The routes of administration are intraperitoneal, intravenous, and anal injection. Wang and colleagues demonstrated that intraperitoneal administration of MSCs is superior to the other two techniques in colitis. They noted that intraperitoneal injection resulted in the highest survival rate of 87.5% (coupled with quick weight gain), nearly absent fecal occult blood at day 3, lowest TNF-*α* and highest IL-10 and TSG-6 levels, highest FoxP3+ cells accumulation, and Ki-67 proliferative repair. However, the engraftment intensity of transplanted MSCs within the colonic tissues and mesentery lymph nodes was high in both intraperitoneal and anal injections [69]. With regard to CRC, MSCs can either promote or inhibit their development and progression. The double-edge activities of MSCs within the CRC microenvironment are expounded below.

## 7. The Double Edge of MSC Activities in IBD-Associated CRC

Mesenchymal cells within the intestine play several roles including providing structural support and maintaining homeostasis. Recent studies have established their crucial role in the development of CRC, and animal model studies have documented their link in the pathogenesis of both colitis-associated cancer and sporadic CRC. The recruitment of bone marrow-derived MSCs and fibrocytes, together with resident mesenchymal cells, activates tumor mesenchymal cells (cancer-associated fibroblasts) [70]. These cancer-associated fibroblasts participate in several processes that result in the promotion of colon tumor development and progression. Wu and colleagues in their assessment of the tumor-enhancing effects of MSCs in CRC report that MSCs greatly enhance CRC progression by encouraging cell migration, proliferation, and colony formation [71]. Further analysis revealed that the cancer progression was via AMPK/mTOR-mediated NF-*κ*B activation. Similar studies also linked the progression of the CRC to IL-6/JAK2/STAT3 signaling, which activated PI3K/AKT signaling [72], and also via direct cell-to-cell contact [73]. TNF-*α*-primed-human-MSCs also promote CRC through the CCl5/*β*-catenin/Slug pathway by increasing activities such as epithelial-mesenchymal transition, cell proliferation, migration, and invasion [74].

Although MSCs have grossly been implicated in the growth, invasion, and metastasis of cancer cells, they still possess a lot of potentials to ameliorate CRC under certain instances [70, 75, 76]. Francesco and colleagues report that MSCs exert a powerful therapeutic function in a colitis-associated CRC by reducing Ki67 through the blockade of the Smad2 signal pathway resulting in lengthened colon and decreased number of tumors [77]. Similarly, another study found that MSCs reduced the number of tumors by preventing their unset but not the sizes of already established ones [78]. Other studies also demonstrate that bone marrow MSCs (bmMSCs) could remiss colitis-associated CRC by inhibiting the phosphorylation of STAT3 with resultant weight gain and reduced expressions of proinflammatory factors [79]. Again, bmMSCs expressed specific cytokines which impeded the proliferation of CRC cells through the inhibition of the PI3K/AKT pathway and the expression of extracellular signal-regulated protein kinase (ERK), when low doses of either X-rays or UV irradiation were administration [80].

In the TME, MSCs exert their immunologic functions by influencing the cytokine secretion of cellular components like APC, NK cells, and T cells giving MSCs dual functional abilities owing to the fact that they could enhance both apoptosis and survival of tumorigenic cells [81]. In the light of this, the therapeutic utilization of MSCs in all forms of CRC appears promising but the method, dosage, complexity of carcinoma, and procedure, among several other factors still need further investigations especially on the phase that available data on the therapy effects of MSCs are controversial.

## 8. Therapeutic Utility of MSC in Experimental IBD

Tissue inflammatory damages and dysregulation of immune responses are the key pathogenic characteristics of IBDs, and MSCs are known to provide an effectual therapeutic impart in inflammatory diseases via the regulation of inflammatory responses, and tissue regeneration based on their differentiation abilities and molecular mechanisms. The MSC reparative effects can be broadly categorized into cytokine regulatory repair and direct cellular engraftment repair as discussed below.

### 8.1. Cytokine Regulatory Repair

IBD is a multifactorial chronic relapsing condition characterized by aberrant systemic and mucosal immune responses against intraluminal antigens, altered microbial factors composition, and compromised mucosal barrier integrity [82]. The mechanism involved in the release of regulatory cytokines by MSCs is a complex system which integrates inflammatory modulators and pathogenic agents with toll-like receptors and other surfaces. These cytokines include IL-4, IDO, IL-10, GATA3, IL-13, TGF-?, and PGEs [3]. One principal focus of MSC cytokine regulatory repair in IBD is to restore the lost balance between proinflammatory Th1/Th17 cells and Treg cells, which is responsible for the recruitment of circulating leucocytes and stimulation of macrophages and B cells in the gut. MSCs effectively migrate and home to the IBD environment and secrete powerful immunoregulatory soluble factors that do not only inhibit the proliferation and function of Th1/Th17 cells but also promote Treg differentiation as well as survival and recovery of injured cells and tissues. This outcome results in increased anti-inflammatory cytokines like TGF-*β*, IL-4, IL-10, IL-11, and IL-13 and decreased inflammatory cytokines like IL-6, IL-12, IL-23, and IL-21 [83].

An additional factor involved in IBD pathogenesis is imbalance of Bax protein (proapoptotic) and Bcl-2 protein (antiapoptotic), which causes defective immune cell apoptosis [84]. MSCs effectively induce T cell apoptosis through the FAS ligand- (FASL-) dependent FAS pathway to attenuate DSS-induced colitis. In this mechanism, FAS-modulated MCP-1 (monocyte chemotactic protein 1) secreted by MSCs recruits T cells for FASL-mediated apoptosis. The apoptotic T cells consequently stimulate macrophages to express high levels of TGF*β*, which in turn lead to upregulation of CD4(+)CD25(+)Foxp3(+) Treg cells and, finally, immune tolerance [85].

In application, a therapeutic efficacy assessment of MSCs by Ahmed and colleagues indicates that genetic expressions of markers of inflammation (IL-23, IFN-*γ*, TNF-*α*, and ICAM-1) within the intestinal mucosa of MSC-treated mice appreciably lowered, resulting in a significant improvement in weight gain, stool condition, and normal histopathology of tissues analyzed [86]. In another work, the amount of Treg cells and the expression of TGF-*β* and IL-10 were upregulated while IL-17 levels rather decreased when MSC-conditioned medium was administered [87]. The outcome was inhibited loss of weight and bleeding, enhanced consistency of feces, and improved disease activity index (DAI), as well as decreased colon inflammation and mucosal degeneration. Similar outcome is observed when activated NOD2 signaling increases the ability of human umbilical cord MSCs (hucMSCs) to inhibit mononuclear cell proliferation via the induction of PGE2 production in colitis mice [26]. Intravenous grafts of bmMSCs also prevent the onset of colitis and increase mice survival time via upregulating the expression of Foxp3+ regulatory T cells in mesenteric lymph nodes [88].

Upregulation in the activation of Th2 cells in UC and Th17/Th1 cells in Crohn's disease (CD) alongside decreased Treg levels are noted in IBD [89]. These stimulated T cells are highly apoptotic resilient due to the disparity of the proteins Bcl-x(L), Bcl-2, and Bax which are proapoptotic and antiapoptotic Bcl-2 family proteins [84]. However, the activities of FasL-Fas cause an intravenously administered bmMSCs to induce the apoptosis of T cell in colitis [85]. Again, MSCs were proven to exert direct inhibitory activities on the antigen-presenting functions of macrophages and dendritic cells, making them immunologically tolerant with increased secretions of IL-10 and heightened induction of Treg in the murine colitis model and other experiments [90, 91]. Moreover, MSCs significantly decrease colonic damages and NF-*κ*B activities, increase IL-10 levels [92], produce TGF-? and VEGF receptor to enhance angiogenesis and cellular damage repair, and restrict B lymphocyte proliferation through the promotion of CD40 expression in colitis [93].

### 8.2. Direct Cellular Engraftment Repair

Irrespective of their sources and routes of administration, human derived-MSCs have been shown to be capable of engrafting into the mesenteric lymph node and inflamed intestine in IBD rodents, with reported tissue persistence time ranging from 3 to 15 days [94–96]. In a study by Fawzy and colleagues, MSCs stimulate colonic repair by differentiating into several cells and dampening the inflammation as compared to the untreated colitis group, which experienced severe ulcerations, distorted crypt architecture, and loss of surface columnar epithelium, among others [97]. In a recent study involving endoscopic submucosal injection of adipose-derived MSC in colitis rats, the MSCs were found in the colon submucosa 24 hours after administration and later gained fibroblastic phenotype properties. These MSCs differentiated into fibroblast, caused less inflammatory infiltrate and almost absent edema [41]. In other applications, a systemic infusion of bmMSCs enhanced the differentiation and proliferation of cells within the intestinal epithelium. This was evident by a significantly increased quantity of Lgr5 and Ki67 in the damaged cells of the colon [94]. Other studies have shown the ability of MSCs to effectively migrate and accumulate in inflamed sites of the colon to participate in tissue repair by differentiating into endothelial cells, vascular smooth muscle cells, pericytes, or epithelial cells and also protect colonic cells against apoptosis. Some of the attributed mechanisms include MSCs differentiating into colonic interstitial lineage cells and producing TGF-*β*1 and VEGF [98], increased TGF-*β* mRNA expression and inhibited Notch signaling [99], and stimulation of resting (G0) cells to enter the cell cycle (G1) [97]. In all these investigations, treatment with MSCs resulted in a suppressed Th17/Th1 cells as well as inhibition of other major immune cells like DC, NK cells, and B cells and boosted the induction of antigen-presenting cells into regulatory-like cells within colonic tissues and mesenteric lymph nodes, along with stimulation of intestinal epithelial cell differentiation and proliferation, decreased systemic proinflammatory chemokines like IFN-*γ*, TNF-*α*, IL-17, and IL-6, and increased anti-inflammatory chemokines like IL-10 [41, 93, 95, 96] as already expounded in cytokine-regulated repair.  Table 1 summarizes some of the experimental studies of MSC-based therapy in IBD.

## 9. Therapeutic Utility of MSC in IBD Clinical Trials

**Figure 3:**
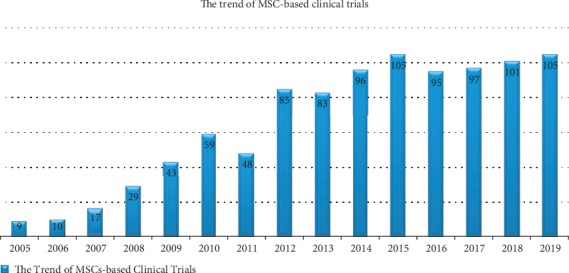
The trend of MSC-based clinical trials within the past 15 years. Each bar represents the total number of registered trials within the year. This indicates an increasing interest in this area with the highest annual registered clinical trials reaching 105 in the years 2015 and 2019, followed by 101 in 2018.

The actively self-renewing, multipotent, and immunosuppressive capabilities of MSCs have attracted increasing clinical investigations on their application in treating several diseases and conditions including immunological disorders like IBD, with increasing trend every year.  Figure 3 presents the general trend of clinical trials involving MSCs as registered in ClinicalTrials.gov from 1^st^ January to 31^st^ December each year for the past 15 years. Using the search word “mesenchymal stem cell”, a total of 982 clinical trials were found registered within this period.

**Figure 4:**
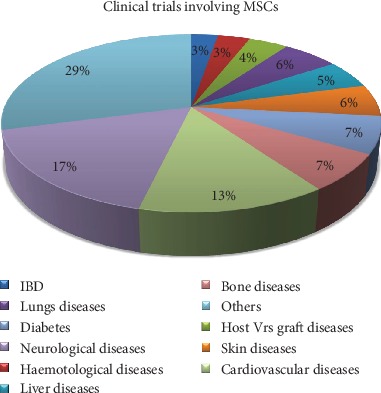
The percentage distribution of MSC-based clinical trials on some selected conditions. Some of the most registered MSC-based clinical trials include neurological diseases (17%), cardiovascular diseases (13%), bone diseases, and diabetes (7% each). IBD contributes approximately 3% to MSC-based clinical trials.

Accordingly, all registered clinical trials including observational and expanded access studies on MSC in all diseases as at January 28^th^, 2020, are 1037. This number is made of both completed and uncompleted studies across various conditions out of which 28 (approximately 3%) are IBD related (Figure 4).

### 9.1. Systemic Infusion

In an expanded phase II trials involving 49 complex cryptoglandular fistulas in CD patients, the administration of combined fibrin glue and two dosages of MSCs obtained from adipose (20 × 10^6^) produced substantially greater efficacy with no adverse events in relation to the MSCs [96]. A long-term result assessment of a previous clinical trial was carried out, during which 41/43 phase II clinical trial clients were monitored within an extra one year. At 24 months, there was complete healing in 21 of 26 clients (80.8%) in modified per protocol analysis and 27 of 36 clients (75.0%) in modified intention-to-treat analysis. Interestingly, there was well-sustained total closure after initial therapy and no adverse events in relation to administered MSCs [102]. On the contrary, although an earlier study on complex perianal fistula treatment showed a promising therapy efficiency with the rate of recovery as high as 71% during a phase II trial, a randomized phase III trial unsuccessfully showed no statistical significance in therapeutic efficacy. Additionally, a long-term retrospective follow-up investigation expanding the phase II trial indicates that there was recurrence of fistulas in a significant proportion of the study population, with only 7/12 initial responders sustaining complete fistula closure [103, 104].

The few investigations conducted on the therapeutic imparts of autologous MSCs administered systemically in luminal IBDs are contradictory. While one of the studies indicated an enhanced clinical outcome with adipose-derived MSCs [102], the other two rather showed that most of the patients had no improvement clinically or even had their conditions worsened when treated with bone marrow-derived MSCs [105, 106]. Interestingly, a better outcome was observed from clinical trials utilizing allogeneic MSCs of umbilical cord or bone marrow, in which disease severity reduced significantly with the occurrence of clinical remission in above half of participants [107, 108]. In one of these studies, 12/15 participants experienced clinical response (80%), 8/15 obtained clinical remission (53%), 7/15 had endoscopic improvement (47%), and one person had serious adverse event (which probably was not the cause of the MSCs given) [108]. The differences observed in these studies could be due to differences in clinical designs applied and the features of the in vitro expanded MSCs used. Although different fistulas have similar pathophysiology, they differ in etiology [102]; hence, the significance of their complexity in such studies could be vital. Altogether, it is highly crucial to assess the efficacy and safety of MSC-based therapies in relation to the cellular origin, dosage, and route of administration and more so explore novel modification techniques to further improve MSC-based clinical applications. It is equally vital to further investigate the complexity of fistulas and properties of in vitro expanded MSCs in relation to specific clinical designs to arrive at effective outcomes of MSC therapy.

### 9.2. Local Inoculation

Although the immune modulatory influence of MSCs is well established in several diseases, the therapeutic potential and efficacy of MSCs directly inoculated into inflammatory large intestines or parentally has not been entirely investigated. Locally inoculated MSCs in several clinical trials indicate that this application in treating patients with perianal fistulas in CD is easy, useful, and safe, usually with no adverse events but significant therapeutic impart [109–112]. Molendijk and colleagues observe stable healing effects through week 24 at which 6/9 (66.7%) patients were completely healed [111]. The efficacy data at week 24 as reported by de la Portilla and colleagues indicated that 69.2% of participants had reduced number of draining fistulas, 56.3% had total/complete fistula closure, and 30% of the participants had all their existing fistula tracts completely closed [113]. Specific outcomes of other clinical trials include enhanced clinical remission with 7/10 complete and 3/10 incomplete closure of fistula openings [109, 110] and 6/8 (75%) fistulas completely healed while the other 2 fistulas partially healed with significantly reduced drainage at week 8 [114]. A similar promising healing success rate of 60% in Crohn's related rectovaginal fistulas is reported in a phase I–IIa clinical trial [115].

Most published clinical trials on fistulising CD were done using adipose tissue-derived MSCs. On the other side, studies on luminal CD have employed systemic administration of human umbilical cord-MSCs (hucMSCs) or bmMSCs to treat the condition. Local inoculation of allogeneic or autologous adipose tissue-MSCs (adMSCs) or bmMSCs have resulted in significant efficacy and reassuring safety in many phase I or II clinical trials. Results were uniformly positive regardless of the origin and irrespective of variations in dosage and schema of injection. However, several researchers have demonstrated the superiority of hucMSCs and adMSCs to bmMSCs in terms of proliferation and differentiation potentials, as well as immunosuppressive functions in experimental studies [116–119]. Other clinical trials focused on further exploring the local inoculation of allogeneic or adMSCs or bmMSCs in fistulising CD are still ongoing [82]. Again, while both allogeneic and autologous MSCs have shown promising efficacy in fistulising CD, allogeneic MSCs rather appear more effective in resolving luminal CD than autologous MSCs [82].  Table 2 presents some of the MSC-based clinical trials in IBD.

## 10. Mesenchymal Stem Cell-Derived Exosomes as Cell-Free Therapy

MSC-derived exosomes contain a great variety of functional proteins, mRNAs, miRNAs, and signaling lipids. As cell-free therapy, they possess improved delivery of exogenous biological particles to target sites [122] and directly into cytosol, circumventing the endosomal/lysosomal pathway [123], consequently increasing transfection efficiency. Due to their small sizes, they are capable of evading the mononuclear phagocytic system's clearance, hence extending their circulatory time for passive targeting of inflammatory and cancerous cells [101]. Compared with their parent cells, these vesicles are more stable and can decrease the inherent safety risks associated with the administration of viable cells, like the risk of occlusion in microvasculature and the risk of possible immune recognition by the host system [124]. Recent development also indicates that exosomes are speedily evolving as potential treatment option for cancer, and potential biomarkers for both the diagnosis and prognosis of cancer and other inflammatory conditions [125]. These special properties among others give MSC-derived exosomes enormous potentials over the parental cell therapy across several conditions including IBD and its associated CRC [101, 126–130].

## 11. Challenges of Mesenchymal Stem Cell Therapy

Although experimental and clinical trial applications of MSCs have demonstrated positive influences in chronic inflammatory and autoimmune disease therapy, their capability to encourage growth of tumors and further metastasis as well as the possible overrated therapeutic potentials still remain matters of concern and consideration in regenerative medicine [131]. Duijvestein and colleagues report that, at weeks 6 after MSC administration, 3 participants had to undergo surgical procedure due to worsening of disease [105]. Similarly, 7/12 patients experienced serious adverse events when a single MSC intravenous infusion was given [106], but upon further investigation, exacerbation of the condition was observed in 5/7 participants while adverse effects in other 2 participants were probably due to the MSC infusions. Locally inoculated allogeneic MSCs in patients suffering from refractory CD and complex fistulas have also been associated with certain adverse reactions like uterine leiomyoma and anal abscess [111–113]. Furthermore, severe adverse events were noticed in moderate to severe UC patients who received multistem therapy made of nonembryonic tissue and adult bone marrow sources [132]. These raise concerns on efficacy and safety among other factors of MSC transplant. The ability of MSCs to get engrafted and/or concentrate at the target site, like homing to the mucosa of the intestine and differentiating into epithelial and other cells to promote direct mucosal damage repair, is highly desirable [3]. However, relatively few MSCs intravenously administered get engrafted at these target sites of injury. Experiments in rodent and dog models have shown that these MSCs get caught-up in lung capillaries during which most are largely cleared, with few going through to the injured target tissue [133]. The therapeutic effects produced by MSCs are also known to be short lived in some studies. Long-term retrospective follow-up investigation expanding a phase II trial indicates that there was recurrence of fistulas in a significant proportion of the study population, with only 7/12 initial responders sustaining complete fistula closure [103, 104].

In addition to observed adverse events, discrepancies in documented results, and poor migration and engraftment of transplanted MSCs, the therapy is also confronted with unconfirmed long-time adverse events. Again, factors like source, type, and preparation of MSCs, route, quantity, duration, and frequency of administration, as well as other disease and microenvironment factor need further clarity. Cellular inherent factors and intestinal microenvironment factors that enhance MSC migration, adhesion, proliferation, and cytokine effects need further exploration. MSC modification or engineering techniques and efficiently combined therapeutic approaches should be highlighted.

## 12. Discussions and Conclusions

MSC therapy has drawn quite a quantum of interest in several research fields because of the capability of these cells to proliferate actively, undergo plastic differentiation, trigger strong immune regulation, exhibit low immunogenicity, and express abundant trophic factor. These powerful inherent properties have ensured the success of MSCs in both in vivo and in vitro experimental setups, to achieve cellular replacement, immunosuppression, and trophic actions, making them desirable in immune interventional and regenerative medicinal therapies. The success story of MSCs in preclinical experiments employing models of induced inflammations, autoimmunity, and cell/tissue damages has created room for clinical trials in several conditions including IBD. Nonetheless, several discrepancies exist between results of available studies. Again, long-term adverse events of MSC usage and the mechanisms of their therapeutic actions largely remain unverified. Moreover, MSC transplant efficacy is highly poor and the detectable period of inoculated MSCs within the inflamed intestine is short. In order to achieve the maximum benefit from MSC therapy, there is the need for more efficient engraftment, differentiation, and proliferation of given MSCs in target tissues. In most cases, engrafted MSCs were so scanty that their dynamics could not be monitored in the target intestinal tissue. Although the probability of achieving full MSC potential remains uncertain, one possible way forward is to experiment the ideal conditions in the intestinal mucosa and stromal tissues under which engrafted MSCs may yield their full therapeutic potentials. As MSC therapy with its curative intention expands in IBD, IBD-associated CRC, and other conditions, it is highly significant to design precise aims and objectives with respect to therapeutic targets, select specific experimental/clinical design, and investigate to clarify the exact mechanisms associated with repair in each clinical/experimental designs towards optimum MSC therapeutic imparts. Several other issues, like MSCs origin and type (allogeneic or autologous), administration procedure (schedule, dosage, route, pretreatment with chemokines or cytokines, etc.), MSC preparation quality control measures (monoclonal/homogenous or polyclonal/heterogeneous), and conditions which cause efficacy in transplantation and ensure suitable cellular differentiation in target locations, must adequately be comprehended. MSC studies should also seek to address issues of patient selection, disease activity, and disease stage in the light of therapeutic efficacy. One study report that a MSC-conditioned medium produced pleiotropic gut trophic factors which enhanced the damage repair of intestinal epithelium [134]. The study therefore concluded that the unearthing of strategies to maximize MSC therapy and MSC-conditioned medium ingredient analysis could create novel opportunities for discovery of drugs and set up the grounds for improved cell therapy in IBD. Again, it is undoubtedly needful to intensely explore the evolving therapeutic potentials of MSC-derived exosomes in IBD and IBD-associated CRC. Exosomes do not only serve as nanocarriers to deliver exogenous biological particles to target sites [135] but are also speedily evolving as potential treatment option and possible biomarker for both prognosis and diagnosis of several conditions [136], and potential cancer vaccines [122]. These tiny lipid bilayer enveloped vesicles possess many merits over other mediators of intercellular communications like hormones, neurotransmitters, and cytokines; in that, while these mediators trigger cells via several separate signals, exosomes could execute several signals concurrently [135].

## Figures and Tables

**Table 1:** Experimental studies of MSC therapy in IBD/IBD-associated CRC.

MSC source/type	Model used	Route of administration	Condition	Vital mechanisms	Outcome	Reference
Adipose/allogeneic	BALB/c mice	Intraperitoneal injection	Colitis in CD	(i) Downregulation of Th1 (ii) Impaired Th1 cell expansion (iii) Induced/activated CD4+CD25+FoxP3+ regulatory T cells	(i) Ameliorated clinical and histopathologic severity of colitis (ii) Abrogated body weight loss, diarrhea, and inflammation (iii) Increased survival	[96]

Umbilical cord	C57BL/6 mice	Intravenous injection	AOM and DSS-induced colitis-associated CRC	(i) Decreased expression of Ki-67 (ii) MSCs secreted TGF-*β* to induce Treg cells from naïve T cells (iii) Activated Smad2 signaling	(i) Longer colon length and decreased tumor numbers (ii) Alleviated pathology of inflammation and inhibited inflammation cytokines (iii) Suppressed development of colitis-associated CRC	[100]

Bone marrow	C57Bl/6 mice	Tail vein or intraperitoneal Injection	TNBS induced colitis	Increased Foxp3+ splenocytes/regulatory T cells in a CD11b+ cell-dependent manner	(i) Improved symptoms of colitis (ii) Improved survival (tail vein injection) (iii) No significant improvement in survival (intraperitoneal inj) (iv) Nearly complete absence of occult blood in feces (v) Inhibited histopathological changes in gut-associated tissue	[88]

Adipose	SD-OFA rats	Endoscopic submucosal injection	TNBS-induced colitis	Recovered Foxp3 and IL-10 mRNA levels	(i) Weight lose recovered (ii) Improved endoscopic score (iii) Significantly recovered colon length	[41]

Adipose	Balb/c mice	Intraperitoneal injection	TNBS and DSS induced colitis	(i) ASCs induce a distinct regulatory activation state of macrophages (ii) High arginase activity and increased production of IL-10 (iii) Immunosuppression of T-cells and macrophages (iv) Activation of cyclo-oxygenase-2	(i) Inhibited colitis reducing mortality and weight loss (ii) Reduced levels of inflammatory cytokines (iii) -Reduced transmural inflammation, mucin-producing goblet cell depletion, epithelial ulceration, disseminated fibrosis, focal loss of crypts, and infiltration of inflammatory cells	[90]

Umbilical cord	Mice	Intravenous injection	TNBS-induced colitis	Down-regulated levels of IL-17, IL-23, IFN-*γ*, and IL-6	(i) Improved clinical and pathological signs of colitis (ii) Effectively ameliorated colitis	[95]

Bone marrow	BALB/c mice	Intravenous injection	TNBS-induced colitis	(i) Activated CD4+CD25+Foxp3+ regulatory T cells (TGF-*β*, IL-10, Foxp3) (ii) Downregulated Th1-Th17-driven autoimmune and inflammatory responses (IL-2, TNF-*α*, IFN-*γ*, T-bet; IL-6, IL-17, ROR*γ*t) (iii) Upregulated Th2 activities (IL-4, IL-10, GATA-3)	(i) Ameliorated clinical and histopathologic severity of colitis, including body weight loss, diarrhea and inflammation (ii) Increased survival (iii) Promoted proliferation of intestinal epithelial cells and differentiation of intestinal stem cells	[94]

Umbilical cord	NOD.CB_17_-Prkdc^scid^/J mice	Tail vein injection	DSS induced colitis	(i) Decreased MPO levels hence reduced neutrophil infiltration (ii) Decreased MMP2 and MMP9 activities	(i) Significantly reduced DAI with attenuated presence of bloody stools, weight loss and colon length. (ii) Reduced inflammation and inflammatory cell infiltration, crypt damage, and edema of submucosa	[101]

Umbilical cord	BALB/c mice	Intraperitoneal injection	TNBS-induced colitis	(i) Increased Tregs and CD5+ B cells and decreased Th1, Th17 cells (ii) -CD5+ B cells inhibited T-cell proliferation and produced IL-10	(i) Increased survival rates, relieved symptoms, and improved macroscopic and histologic scores (ii) Alleviated induced colitis	[93]

Umbilical cord		Intraperitoneal injections	DSS or TNBS-induced colitis	(i) Increased IL-10 and Treg cells, and decreased inflammatory cytokines (ii) NOD2 signaling suppressed mononuclear cell proliferation by inducing production of PGE2. (iii) Reduced MPO activity and infiltration of CD4+ and CD11b+ cells	(i) Reduced severity of colitis and recovered loss of body weight and decreased mortality (ii) -Abrogated colitis-induced lethality, improved DAI, restored colon length (iii) -Reduced colon mucosal destruction and edema	[26]

Experimental observations obtained from MSC-based therapy in IBD and its associated CRC. In each observation, route of administration, type of IBD as well as vital mechanisms, and final outcome of the therapy are outlined.

**Table 2:** Clinical trials of MSC-based therapy in IBD.

MSC source/type	Type of trial	Route of administration	Condition	Outcome	Reference
Adipose/allogeneic	Phase 3 randomized, double-blind controlled trial	Intralesional injection	Perianal fistulas in CD	(i) Significant clinical remission (ii) Improved PDAI	[112]

Bone marrow/allogeneic	Efficacy and safety study	Intravenous injection	UC	(i) Powerful immunomodulatory effects (ii) Reduced activity of autoimmune inflammation and stimulated reparative process in the intestinal mucosa, hence increasing the duration of remission, reducing the risk of recurrence of disease, and reducing the frequency of hospitalizations	[120]

Bone marrow/allogeneic	Double-blind, placebocontrolled study	Intralesional injection	Perianal fistulas in CD	(i) Promoted healing of perianal fistulas (ii) Improved PDAI	[111]

Umbilical cord	Non-randomized safety and therapeutic efficacy study	Intravenous infusion	Moderate to severe UC	(i) 30/36 patients treated with MSC showed good response (ii) Diffuse and deep ulcer formation and severe inflammatory mucosa were improved markedly (iii) -During the follow-up, the median Mayo score and histology score were decreased while IBDQ scores significantly improved	[121]

Bone marrow/allogeneic	Phase 2, open-label, multicenter study	Intravenous infusions	Luminal CD	(i) Reduced CDAI and CDEIS scores (ii) Significant clinical remission	[108]

Adipose/autologous	Long-term sustained response assessment	Submucosal fistula injection	Fistulas in CD	(i) High proportion of complete fistula closure (ii) Sustained efficacy and safety	[102]

Adipose/autologous	Prospective phase I clinical trial	Fistula inoculation	Fistula in CD	(i) 6/8 fistulas completely healed. The other 2 with incomplete closure of the external opening	[114]

Bone marrow/autologous	Phase I safety and feasibility study	Intravenous injection	Refractory CD	(i) Significantly reduced CDAI and CDEIS scores (ii) While 3/10 showed clinical response, another 3/10 required surgery due to disease worsening	[105]

Adipose/allogeneic	Multicenter phase I/IIa clinical trial	Intralesionally injection	Perianal fistula in CD	(i) 56.3% of the patients achieved complete closure of the treated fistula (ii) MRI score of severity showed marked reduction at week 24	[113]

Adipose/allogeneic	Phase I-IIa clinical trial	Intralesional injection	Crohn's-related rectovaginal fistula	60% of patients achieved a complete healing	[115]

A summary of clinical trials obtained from MSC-based therapy in IBD. In each observation, route of administration, type of IBD, and final outcome of the therapy are outlined.
